# Retrospective Study for the Clinical Evaluation of a Real-Time PCR Assay with Lyophilized and Ready-to-Use Reagents for *Streptococcus agalactiae* Detection in Prenatal Screening Specimens

**DOI:** 10.3390/diagnostics12092189

**Published:** 2022-09-09

**Authors:** María Paz Peris, Gloria Martín-Saco, Henar Alonso-Ezcurra, Cristina Escolar-Miñana, Antonio Rezusta, Raquel Acero, Ana Milagro-Beamonte

**Affiliations:** 1 Health Research Institute Aragón, 50009 Zaragoza, Spain; 2 Department of Animal Pathology, Faculty of Veterinary, University of Zaragoza, 50009 Zaragoza, Spain; 3 Miguel Servet University Hospital, Microbiology, 50009 Zaragoza, Spain; 4 Department of Microbiology, Paediatrics, Radiology, and Public Health, Faculty of Medicine, University of Zaragoza, 50009 Zaragoza, Spain; 5 Department of Animal Production and Food Science, Faculty of Veterinary, University of Zaragoza, 50009 Zaragoza, Spain; 6 Department of Design and Manufacturing Engineering, School of Engineering and Architecture, University of Zaragoza, 50009 Zaragoza, Spain; 7 Instituto de Investigación en Ingeniería de Aragón (I3A), University of Zaragoza, 50009 Zaragoza, Spain

**Keywords:** *Streptococcus agalactiae*, molecular diagnosis, clinical validation, GBS culture, rapid diagnostic test

## Abstract

*Streptococcus agalactiae* is a leading cause of sepsis and meningitis in newborns and young infants. Screening programs and intrapartum antibiotic prophylaxis have reduced early neonatal onset of disease. The aim of this study was to evaluate a molecular assay with lyophilized and ready-to-use reagents: VIASURE^®^ *Streptococcus B* Real Time PCR detection kit (CerTest Biotec) (Viasure qPCR assay) compared to both the GBS culture and a molecular assay with separated and frozen reagents: Strep B Real-TM Quant (Sacace Biotecnologies^®^) (Sacace qPCR assay). A total of 413 vaginal–rectal swabs from women between the 35th and 37th weeks of pregnancy were processed. GBS culture was firstly achieved through Granada medium and Columbia CNA agar at 35 °C in aerobic conditions. Then, nucleic acid extraction was performed for subsequent molecular analysis using both commercial assays. Discordant results were resolved via bidirectional Sanger sequencing. Viasure qPCR assay clinical sensitivity was 0.97 (0.92–0.99) and specificity 1 (0.98–1). This retrospective study demonstrated the good clinical parameters and the strong overall agreement (99.3%) between the Viasure qPCR assay and both reference assays. Finally, the added value observed of the assay under study was the stabilized and ready-to-use format, reducing the number of time-consuming steps, permitting the storage at room temperature, facilitating transport, being environmentally respectful, and reducing additional costs.

## 1. Introduction

*Streptococcus agalactiae*, also known as Group B Streptococcus (GBS), colonizes (transiently, intermittently or persistently) the gastrointestinal and/or genital tract of 15% to 40% of healthy adults, the rectum being the reservoir [1].

Colonization in pregnant women is distributed worldwide with an estimation of 11% to 35%, directly related to socioeconomic factors [2]. European countries range from 6.5% to 36% [3], and data published in Spain estimate a 12% to 20% GBS prevalence [4,5].

Due to vertical transmission, two different clinical syndromes are identified in infants according to onset age: early-onset disease presenting mainly sepsis during the first week of life, and late-onset disease affecting infants until three months of age, with bacteriemia and/or meningitis [6,7].

Screening programs and intrapartum antibiotic prophylaxis (IAP) have reduced early neonatal onset disease. Centers for Disease Control and Prevention (CDC) recommends antenatal GBS screening in vaginal–rectal samples between the 35th and 37th weeks of pregnancy, suggesting IAP only for positive cases [8].

Linked to immune and metabolic alterations, antibiotics are important disruptors of the intestinal microbiota establishment at the beginning of life [9,10]. IAP is known to negatively affect the gut microbiota composition, mainly affecting the Actinobacteria, Bacteroidetes, Proteobacteria, Firmicutes and Bifidobacterium relative proportions [11,12,13]. A correct establishment of this microbiota in the early days of life is a determinant for the health of the individual by reducing the later development of chronic diseases, such as obesity, allergies, infections, and inflammatory or brain disorders [14].

Culture is the recommended screening method. However, as GBS culture requires a turnaround time of 18–48 h, alternative methods such as nucleic acid amplification tests (NAAT) are proposed to be performed intrapartum and in uncontrolled pregnancies to avoid the use of unnecessary antibiotics [4].

In this context, the aim of this study was to evaluate the VIASURE^®^ *Streptococcus B* Real Time PCR Detection kit (Viasure qPCR assay) for the detection of GBS through real-time PCR. This product was compared with the GBS culture, which is the recognized gold standard for the clinical diagnosis of this microorganism [4].

Generally, commercially available molecular diagnostic assays require freezing for proper stability and storage of their reagents. However, the assay under evaluation does not require freezing since all the necessary reagents are in a lyophilized and ready-to-use format. Therefore, this qPCR assay was also compared to a commercially available molecular biology assay: Strep B Real-TM Quant from Sacace Biotechnologies^®^ (Sacace qPCR assay).

## 2. Materials and Methods

### 2.1. Study Design and Ethics Approval

This evaluation was conducted in the Clinical Microbiology Laboratory from the Miguel Servet University Hospital (Zaragoza, Spain). It was a comparative, retrospective, observational study where GBS culture and the Sacace qPCR assay were considered the reference methods to calculate clinical sensitivity, specificity, negative and positive predictive values, and overall agreement for Viasure qPCR assay.

The algorithm used for the clinical analysis was: (i) results from GBS culture and Sacace qPCR assay were considered reference values; (ii) discrepant results were resolved by sequencing.

The project follows the requirements of Spanish Policy for Biomedical Research 14/2007, of July 3. The use of all data and samples was approved by the research ethics committee of Aragon (Comité de Ética de la Investigación de la Comunidad Autónoma de Aragón: CEICA) (Project license: PI21/364, date of approval: 28 July 2021) in collaboration with Biobanco del Sistema de Salud de Aragón (SA21-20).

### 2.2. Participants and Samples

From May 2021 to August 2021, a total of 1016 vaginal-rectal specimens were analyzed in the Laboratory by culture from women between the 35th and 37th weeks of pregnancy and processed according to Spanish Society of Infectious Diseases and Clinical Microbiology (SEIMC, 2015) procedures [4], 108 being positives and 908 negatives. For this study, 108 culture-positive specimens and 305 culture-negative specimens randomly selected (with Excel RAND function) were used, being a total of 413 samples.

Once specimens arrived at the Laboratory, culturing was first performed and the remnant liquid medium was stored at 4 °C for a maximum of 7 days prior to molecular analysis. These samples were obtained through the Biobanco del Sistema de Salud de Aragón, which ensured their good condition until the molecular study.

### 2.3. Test Methods 

#### 2.3.1. GBS-Culture: Routinary Laboratory Method

Based on SEIMC, 2015 [4] procedures, swab specimens were cultured with automation systems WASP^®^ (Walk Away Specimen Processor) (Copan, Brescia, Italy) on the specific, selective and differential medium Granada (Oxoid^®^, Hampshire, UK) at 35 °C in anaerobic conditions by covering the inoculated plate with a cover slide. Simultaneously, swabs were cultured on the selective medium for Gram-positive Columbia CNA (Colistin and Nalidixic Acid) agar (bioMerieux^®^, Marcy-l’Étoile, France) at 35 °C in aerobic conditions, to look for *β*-haemolytic colonies. If no GBS presence was obtained after 48 h incubation, the sample was considered negative. The isolates’ identification was confirmed by matrix-assisted laser desorption ionization–time of flight (MALDI-TOF) mass spectrometry (MALDI Biotyper^®^ sirius System, Bruker Daltonics, Billerica, MA, USA) using the MBT Compass Library Rev L 2020 and the software version MBT Compass, and rapid agglutination as an alternative technique.

#### 2.3.2. NAATs Assays: VIASURE^®^ Streptococcus B Real-Time PCR Detection Kit (CerTest Biotec) and Strep B Real-TM Quant (Sacace Biotechnologies^®^)

DNA was extracted from 200 μL of the remnant vaginal–rectal specimens using automated DNA extractor magLEAD 6gC (Precision System Science^®^ Co., Ltd., Matsudo, Japan) in accordance with the manufacturer’s instructions with a sample processing time of 26 min. All nucleic acid extractions were eluted in 60 μL final volume. Then, DNAs were stored properly at −20 °C until used for molecular analysis.

Viasure qPCR assay amplification was performed in a final reaction volume of 20 μL and using the DTprime Real-Time Detection Thermal Cycler instrument (DNA Technology, Moscow, Russia) following the standard manufacturer’s instructions with 90 min duration. The thermal profile used was: 1 cycle at 95 °C for 2 min for polymerase activation; 45 cycles at 95 °C for 10 s and at 60 °C for 50 s for denaturation and annealing/extension. Fluorescence was measured at the end of the annealing step of each cycle. A positive control and a negative control provided by the kit were used in each run.

The results were interpreted with RealTime_PCR v7.9. Certest BioTec software, with 40 Ct as the low detection limit. The target amplification region was the *cfb* gene conserved region, and the analytical sensitivity of the kit was reported to be ≥ 10 DNA GBS copies per reaction. The batch used was GBS1XXL-008, expiry date: 03/2023. All necessary reagents were lyophilized and ready-to-use (containing a mix of enzymes, primers, probes, buffer, dNTPs, stabilizers and internal control) in the qPCR tube. This allows stability from a 2 °C to 40 °C temperature range.

Regarding Sacace qPCR, assay amplification was performed in a reaction volume of 25 μL and using the CFX96™ real-time PCR system instrument (Bio-Rad^®^ Laboratories, Marnes-la-Coquette, France) with a 105 min duration. The thermal profile used was: 1 cycle at 95 °C for 15 min; 5 cycles at 95 °C for 5 s, 60 °C for 20 s and 72 °C for 20 s; 40 cycles at 95 °C for 40 s and 60 °C for 40 s (fluorescence measurement) for denaturation and annealing/extension. A positive control and a negative control provided by the kit were used in each run.

The fluorescence threshold was automatically adjusted by the CFX Manager™ software. In accordance with the manufacturer’s instructions, 35 Ct was considered the low detection limit. The target amplification region was a conserved gene region not specified in the protocol, and the analytical sensitivity of the kit was reported to be not less than 300 copies/mL. The batch used in this study was 20G21.L364, expiry date: 07/2022. Reagents were presented in three separated tubes at −20 °C and transport required cold packaging.

Both NAATs were performed on the same day and testing was repeated once if invalid results were obtained in the initial test.

### 2.4. Discrepant Results Resolution: Bidirectional Sanger Sequencing

Samples with discrepant results among culture, Viasure qPCR assay and Sacace qPCR assay were sequenced. Briefly, a 150 pb conserved region of the *cfb* gene was amplified using the primers previously described [15]: forward primer 5′-TTCACCAGCTGTATTAGAAGTACATGC-3′ and reverse primer 5′-CCCTGAACATTATCTTTGATATTTCTCA-3′. The non-purified PCR product together with the PCR forward and reverse primers (both at 25 pmol) were sent to STAB VIDA, Lda (Caparica, Portugal) for purified PCR amplicons and bidirectional Sanger sequencing. Obtained sequences were assembled and edited manually by means of the CodonCode Aligner software (CodonCode Corporation, Dedham, Massachusetts, USA). A BLAST search (https://blast.ncbi.nlm.nih.gov/Blast.cgi accessed on 15 November 2021) was performed using the obtained nucleotide sequences for species classification.

### 2.5. Data Collection and Analysis

The data was collected in an Excel file including GBS-culture and both NAATs assays results. The GBS culture method and Sacace qPCR assay were considered the reference assays to calculate clinical sensitivity, specificity, negative and positive predictive values, and overall agreement between techniques (with 95% confidence intervals) using the MetaDisc v1.4 freeware software [16]. Clinical sensitivity, specificity, negative and positive predictive value calculations were based on the following formulas: sensitivity = true positive samples/(true positive samples + false negative samples); specificity = true negative samples/(false positive samples + true negative samples); positive predictive value = true positive samples/(true positive samples + false positive samples); negative predictive value = true negative samples/(false negative samples + true negative samples). The minimum sample size was calculated with WinEpi 2.0 (http://www.winepi.net/winepi2/ accessed on 2 July 2021) [17] with the estimate proportion (random sampling and perfect diagnostic) option. From an unknown population size, and using a calculation based on binomial distribution, the minimum sample size calculated was 209 individuals to analyze an estimated proportion of 12% with an accepted error (or precision) of 5% and a confidence level of 95%.

## 3. Results

From May 2021 to August 2021, the GBS-positive prevalence (with 95% confidence interval) from the 1016 specimens (108 positives and 908 negatives) studied by culture in the laboratory was 10.6% (Confidence interval: 8–12).

From the 413 samples used in this study, all the 108/413 (26.1%) culture-positive specimens were positive by both NAATs. Viasure qPCR assay yielded 112/413 (27.1%) positive results and Sacace qPCR assay 115/413 (27.8%) positive results (Table 1).

Viasure qPCR assay detected GBS presence in four samples not previously detected by culture (ID 026, 197, 252 and 257). Sacace qPCR assay detected GBS presence in these four samples and in three other samples not detected by either culture or Viasure qPCR assay, representing a total of seven discrepant samples (ID 026, 197, 252, 255, 257, 327 and 333).

After sequencing, all generated sequences with suitable sizes were blasted for species classification confirming GBS presence. The highest percent identity for a set of aligned segments to the same subject sequence, ranged between 94.4% and 100%. Discrepancies among techniques are shown in Table 2.

After sequencing and using the algorithm proposed in this study, 115/413 specimens (27.8%) were defined as true positives and 298/413 (72.2%) as true negatives.

The GBS detection rate was 26.1% (108/413) for GBS-culture, 27.1% (112/413) for Viasure qPCR assay and 27.8% (115/413) for the Sacace qPCR assay. No false negative results were obtained by Sacace qPCR assay; however, GBS-culture and Viasure qPCR assay yielded seven and three false negative results, respectively. No false positive results were observed by either technique (Table 3).

Finally, an increased detection rate from 3.7% to 6.4% was observed with Viasure and Sacace qPCR assays, respectively, when compared to GBS culture.

## 4. Discussion

This retrospective study demonstrated the good clinical sensibility and specificity of a molecular assay with lyophilized and ready-to-use reagents (Viasure qPCR assay) compared to both the GBS-culture and another NAAT with separated and frozen reagents (Sacace qPCR assay).

Both NAATs were able to detect all culture-positive specimens, even detecting GBS-positive specimens not detected by the culture method. Four samples were positive by Viasure qPCR assay but negative by culture, and seven samples were positive by Sacace qPCR assay but negative by culture. The mean Ct values obtained in these specimens (31.8, SD: 2.5 and 30.4, SD:2.2) suggested moderate levels of nucleic acids. These results demonstrated higher sensitivity than culture, as previously reported [18].

The GBS-positive rate increment has been observed in previous studies which vary by type of NAAT, targeted gene and study population [19,20,21]. In this study, an increased detection rate of 3.7% to 4.6% was observed with Viasure and Sacace qPCR assays, respectively, when compared to culture. This agrees with other commercial NAATs studies, for example, BD MAX™ [22,23,24], Illumigene^®^ [23,24], the BD GeneOhm™ [23], AmpliVue^®^ GBS Assay (Quidel) [24], Hologic Panther Fusion, Luminex Aries GBS, and Cepheid Xpert [18]. All of them have been demonstrated to be less hands-on, have a faster time to result, and potentially have greater sensitivity than culture methods. This leads to a consideration of NAATs as the new gold standard for intrapartum GBS screening [18].

Comparing the two molecular methods used in this study, Viasure qPCR assay reagents are lyophilized in a stabilized format in the qPCR reaction tube (containing a mix of enzymes, primers probes, buffer, dNTPs, stabilizers and internal control) allowing greater stability from a 2 °C to 40 °C temperature range. This leads to less manipulation and, therefore, less handle error. To the authors’ knowledge, Viasure qPCR assay is the only product that presents this lyophilized and ready-to-use format. However, Sacace qPCR assay reagents are separate in a liquid state, requiring more hands-on time. Liquid reagents’ state makes it necessary to transport between 2 °C and 8 °C and store at −20 °C. A positive aspect of the Sacace qPCR assay is the endogenous internal control amplification which is apart from the internal reaction control. It allows not only the controlling the analysis steps, but also estimating sample handling and storage.

Culture remains the reference diagnostic method for GBS colonization detection. This requires 18–48 h, being feasible only for antepartum screening and excluding women with preterm delivery (<35 weeks gestation) and uncontrolled pregnancies. The time required to perform Viasure and Sacace qPCR assays was approximately two hours each, demonstrating that a rapid tool such as qPCR could be implemented to avoid unnecessary antibiotics treatments. This would elude the modification of the microbiota establishment at the beginning of life caused by antibiotics [18]. On the other hand, it is remarkable that most remaining cases of early onset occur in newborns whose mothers had a negative result in the screening for GBS colonization at 35 to 37 weeks of gestation [25].

The real-time PCR technique has high specificity, wide detection range, speed in the visualization of the product, and can detect differences of a single DNA copy [26]. Therefore, qPCR is a good candidate to be considered as a Rapid diagnostic test. According to the ASSURED requirements established by the WHO, Rapid tests must be affordable, sensitive, specific, user-friendly, rapid and robust, equipment-free, and deliverable to end users. Furthermore, they should be environmentally safe and low cost [27,28].

Regarding the commercial NAATs performance assay time, Relich 2018 [29] evaluated temporal aspects, including the hands-on time and total turnaround time, of ARIES^®^ GBS, BD MAX™ GBS, Illumigene^®^ GBS and Xpert^®^ GBS LB GBS molecular diagnostic tests. The latter was the fastest, requiring less than 1 h 12 min from sample manipulation to result reporting for one, six, and twelve samples. The slowest was the BD MAX™GBS assay when 12 samples were tested (time: 2 h 10 min). In regard to our study, automated DNA extractor magLEAD 6gC (Precision System Science^®^ Co., Ltd.) has a sample processing time of 26 min. However, not all molecular biology laboratories have automated DNA extractors. Alternative methods such as manual extraction or column extraction kits could be used, increasing the time to obtain a result because of the handling time. Even so, due to the easy handling, besides the 90 min thermocycler protocol duration, Viasure qPCR assay could achieve the characteristics needed to be considered a Rapid diagnosis test for implementation intrapartum.

NAATs for GBS diagnosis evaluated in other studies [18,22,23,24,29] do not require prior DNA extraction, but specific platforms for each molecular technique are necessary. This makes it non-equipment-free; however, both Viasure and Sacace qPCR assays allow GBS detection in different instruments, taking advantage of thermocyclers from the laboratories themselves. Furthermore, the Viasure qPCR assay allows different available formats to adjust to the laboratory diagnostic capacity: plates in a low (0.1 mL) and high (0.2 mL) profile, 2 mL tubes, and eight-well individual strips in a low and high profile.

On the other hand, Sacace qPCR assay allows the bacterium quantification. However, this characteristic is not remarkable since, both with a large and small number of bacteria present, the treatment would be the same [30].

Molecular methods are limited in GBS antibiotic resistance gene detection, and to the authors’ knowledge, it has not yet been incorporated into the PCR primer set. This could enable important information in the context of penicillin and beta-lactam allergies [31,32]. Meanwhile, GBS antibiotic resistance detection via microbiological methods could be recommended after qPCR GBS presence confirmation. This would reduce time and resources.

This study has some limitations. Our results would be more accurate if the study were prospective and multicentric rather than retrospective and single-center. Furthermore, no enrichment step is added in our laboratory and Granada medium is used as an alternative. This could probably improve GBS culture detection rate.

## 5. Conclusions

The findings obtained in this study lead to considering the implementation of NAATs assays intrapartum to include GBS diagnosis in women with preterm delivery <35 weeks’ gestation and uncontrolled pregnancies. Furthermore, this retrospective study demonstrates the good clinical parameters and the strong overall agreement between the VIASURE^®^ *Streptococcus B* Real Time PCR detection kit (CerTest Biotec) compared to both the GBS culture (routinary used in the Laboratory) and Strep B Real-TM Quant (Sacace Biotecnologies^®^). The added values observed of the stabilized and ready-to-use format, are through the time-consuming step reduction, permitting storage at room temperature, facilitating transport, being environmentally respectful, and reducing additional costs. Thus, it could achieve the characteristics needed to be considered a Rapid diagnosis test for implementation intrapartum.

## Figures and Tables

**Table 1:** Comparison of Viasure qPCR assay results to GBS culture and Sacace qPCR assay results.

		GBS-Culture			Sacace qPCR Assay		
		Positive	Negative	Total	Positive	Negative	Total
**Viasure qPCR assay**	Positive	108	4	112	112	0	112
	Negative	0	301	301	3	298	301
	Total	108	305	413	115	298	413

**Table 2:** Discrepancies in GBS detection among GBS culture, Viasure qPCR and Sacace qPCR assays, and sequencing results.

ID	GBS-Culture	Viasure qPCR Assay	Sacace qPCR Assay	Sequencing Fw / Rv % Ident.
026	Negative	Positive Ct = 28.2	Positive Ct = 26.9	100/99.1
197	Negative	Positive Ct = 32.9	Positive Ct = 30.2	99/99.1
252	Negative	Positive Ct = 33.2	Positive Ct = 31.8	99.1/98.2
255	Negative	Negative	Positive Ct = 30.1	100/100
257	Negative	Positive Ct = 33	Positive Ct = 33.0	96.4/100
327	Negative	Negative	Positive Ct = 33.8	98.3/99.1
333	Negative	Negative	Positive Ct = 32.9	100/99.1

Fw/Rv % ident: forward and reverse primers percent identity.

**Table 3:** Clinical validation results (95% confidence interval) after the study of the discordant samples.

	TP	TN	FP	FN	SE	SP	PPV	NPV	OA%
Viasure qPCR assay	112	298	0	3	0.97 (0.92–0.99)	1 (0.98–1)	1 (0.96–1)	0.99 (0.97–0.99)	99.3 (97.9–99.8)
Sacace qPCR assay	115	298	0	0	1 (0.96–1)	1 (0.98–1)	1 (0.95–1)	1 (0.98–1)	100 (99.1–100)
GBS culture	108	298	0	7	0.93 (0.88–0.97)	1 (0.98–1)	1 (0.95–1)	0.97 (0.95–0.98)	98.3 (95.5–99.2)

TP: true positive; TN: true negative; FP: false positive; FN: false negative; SE: sensitivity; SP: specificity; PPV: positive predictive values; NPV: negative predictive values; OA%: overall agreement percentage.

## Data Availability

Not applicable.
